# Autophagy in Obesity and Type 2 Diabetes: Beyond the Protective Paradigm

**DOI:** 10.1007/s13679-026-00716-5

**Published:** 2026-05-08

**Authors:** M. Elena Angarita-Plánchez, Paula Sánchez-Rodríguez, Ana M. Múnera-Rodríguez, Camila Leiva-Castro, Juan Manuel Benítez-Márquez, Icíar Reina-Pérez, Francisca Palomares, Soledad López-Enríquez

**Affiliations:** 1 Department of Medical Biochemistry, Molecular Biology and Immunology, Faculty of Medicine, University of Seville. Av. Sánchez Pizjuan S/N, 41009 Seville, Spain; 2 Institute of Biomedicine of Seville (IBiS), Virgen del Rocío University Hospital, Virgen Macarena University Hospital, University of Seville, CSIC, Seville, Spain

**Keywords:** Autophagy flux, Mitophagy, Obesity, Type 2 diabetes, Biomarkers, Therapeutic targets

## Abstract

**Purpose of Review:**

This review examines the current evidence on autophagy dysregulation in obesity and type 2 diabetes mellitus (T2DM), with particular emphasis on its tissue-specific nature and implications for clinical translation.

**Recent Findings:**

Recent human and preclinical evidence indicates that autophagic alterations in metabolic disease are not uniformly suppressed but vary according to tissue type and disease stage. In adipose tissue, liver, skeletal muscle, pancreatic β cells, and immune cells, dysregulated mTORC1-AMPK signaling, defective mitophagy, and impaired lysosomal function contribute to insulin resistance, ectopic lipid accumulation, and metaflammation. However, most human studies rely on static markers such as LC3, p62, and Beclin-1, which do not reliably reflect dynamic autophagic flux. Recent advances, including organelle-specific biomarkers, ex vivo functional assays, and circulating exosomal cargo, offer new translational opportunities, although standardization remains limited.

**Summary:**

Autophagy in metabolic disease represents a context-dependent maladaptation rather than a uniformly protective pathway. Future progress will depend on harmonized biomarker panels, functional assessment of autophagic flux in humans, and integration with metabolic phenotyping to enable precision-based therapeutic strategies.

## Introduction

The sustained global increase in obesity has solidified its role as a major risk factor for metabolic diseases, particularly type 2 diabetes mellitus (T2DM) [1], which highlights the need for a deeper understanding of the molecular links between excess adiposity and metabolic dysfunction. Autophagy is now recognized as a key regulator of immune and inflammatory signaling, coordinating cellular stress responses with cytokine production, inflammasome activity, and immune cell homeostasis [2]. In metabolic disorders, this immunoregulatory function intersects with nutrient overload and organelle stress; however, it remains unresolved in humans whether autophagic alterations represent causal drivers of disease or secondary adaptative responses.

Beyond its role in proteostasis, autophagy regulates immune and inflammatory pathways, linking metabolic stress with the low-grade chronic inflammation characteristic of obesity and T2DM [3, 4]. This persistent inflammatory state, termed metainflammation, underscores the close relationship between metabolic dysregulation and insulin resistance [5]. Evidence indicates that obesity and insulin resistance are accompanied by impaired autophagic outflow and disrupted regulatory signaling, contributing to the pathogenesis of T2DM and broader metabolic alterations [6, 7]. These observations have fuelled interest in pharmacological and lifestyle interventions capable of modulating autophagy as potential therapeutic strategies [8].

Although several recent reviews have addressed the role of autophagy in obesity and T2DM. Most research has focused on basic mechanisms in experimental models or isolated clinical observations, without providing an integrated translational framework. Current articles frequently conceptualize autophagy as a uniformly protective pathway and rely heavily on static biomarkers, thereby limiting their applicability to patient monitoring in real-world settings and to the design of targeted interventions.

In this study, we move beyond this protective paradigm by synthesizing tissue-specific and stage-dependent alterations in autophagy in human obesity and T2DM and by proposing an operational panel that integrates tissue, blood, and exosome-derived markers with functional flow assays and metabolic readouts. This approach is specifically designed to bridge mechanistic understanding with clinical decision-making, highlighting how autophagy-centered biomarker circuits could be incorporated into future interventional trials and routine risk stratification.

Importantly, while previous reviews have described tissue-specific alterations in autophagy and have highlighted the limitations of static markers, they have not provided a structured framework for clinical translation. The present work addresses this gap by delivering an integrated and actionable perspective that bridges mechanistic insights with clinical applicability. Specifically, this review: (a) reconciles apparently conflicting evidence regarding autophagy activity across tissues and disease stages, (b) critically evaluates biomarkers within the context of autophagic flux rather than static abundance; and (c) proposes a structured operational framework combining tissue-level, circulating, and functional readouts aligned with metabolic phenotyping.

## Autophagy Framework Relevant to Metabolic Disease

Autophagy is an evolutionarily conserved lysosomal degradation pathway that maintains cellular proteostasis and metabolic homeostasis through the recycling of proteins, organelles, and lipid substrates. Three principal forms are distinguished: microautophagy, macroautophagy, and chaperone-mediated autophagy (CMA). The molecular basis, regulatory nodes, and functional modalities of these processes have been extensively reviewed elsewhere, including by our group in the context of inflammatory diseases [2]. Here, we summarize the most relevant aspects for metabolic disease.

Macroautophagy (hereafter referred to as a autophagy) is the principal form implicated in obesity and T2DM. It involves the sequestration of cytoplasmic cargo within double-membrane autophagosomes, which subsequently fuse with lysosomes for degradation [9]. Canonical initiation is governed by nutrient- and energy-sensing pathways, particularly the inhibition of mammalian target of rapamycin complex 1 (mTORC1) and activation of AMP-activated protein kinase (AMPK), which converge on the ULK1/2 complex together with its cofactors (ATG13, FIP200, ATG101) to trigger the phagophore formation [10–12]. Once activated, this complex promotes phagophore nucleation through the class III PI3K-Beclin1 complex [13], while elongation and closure depend on the ATG5-ATG12-ATG16L1 conjugation system and the lipidation of microtubule-associated protein (LC3) light chain 3, specifically the conversion of LC3-I to LC3-II, which is widely regarded as a classic marker of autophagosome formation [12, 14]. Charge selectivity is mediated by adaptor proteins such as p62/SQSTM1, which bind ubiquitinated proteins to LC3-II, thereby facilitating substrate selection [15–17]. Finally, autophagosomes mature and fuse with lysosomes through factors such as RAB7 and SNARE proteins, enabling lysosomal degradation of their contents and recycling of metabolic products [9, 18]. Detailed mechanistic descriptions are provided in previous reviews [2, 6, 19].

In metabolic tissues, autophagy integrates nutrient sensing with inflammatory signaling and mitochondrial quality control pathways. In obesity, impaired autophagic flux in adipocytes and adipose tissue macrophages promotes adipocyte hypertrophy, endoplasmic reticulum (ER) stress, mitochondrial dysfunction, and activation of inflammatory platforms such as the NLRP3 inflammasome, thereby reinforcing meta-inflammation [20]. In T2DM, autophagy is essential for preserving pancreatic β cell function and viability, as well as maintaining mitochondrial integrity in the liver and skeletal muscle. Autophagic dysfunction leads to progressive β-cell loss, oxidative stress, mitochondrial damage, and ultimately insulin resistance [21]. Furthermore, insulin resistance alters lipid metabolism, promoting ectopic lipid deposition and creating a cellular environment overloaded with metabolic and inflammatory stress [22].

Thus, obesity and T2DM share a bidirectional pathogenic axis of autophagic dysfunction. Excess nutrients, lipotoxicity, and chronic inflammation suppress autophagy in obesity, exacerbating insulin resistance and facilitating progression to T2DM. Conversely, glucotoxicity and mitochondrial damage in T2DM further impair macroautophagic capacity, establishing a vicious cycle of metabolic and inflammatory deterioration [6, 8, 19].

It should be noted that much of the mechanistic understanding of autophagy regulation derives from in vitro systems and animal models of acute stress. Therefore, extrapolation to chronic human metabolic diseases requires caution.

## Molecular Modulation of Autophagy: from Physiological Regulation to Metabolic Maladaptation

### Autophagy in Metabolic Homeostasis

Autophagy is a dynamic and highly regulated process that responds to nutrient availability, hormonal signals, and inflammatory mediators, functioning as a metabolic sensor. During fasting or energy stress, this mechanism is activated to provide metabolic substrates, recycle intracellular components, and preserve cellular homeostasis [23, 24].

**Fig. 1:**
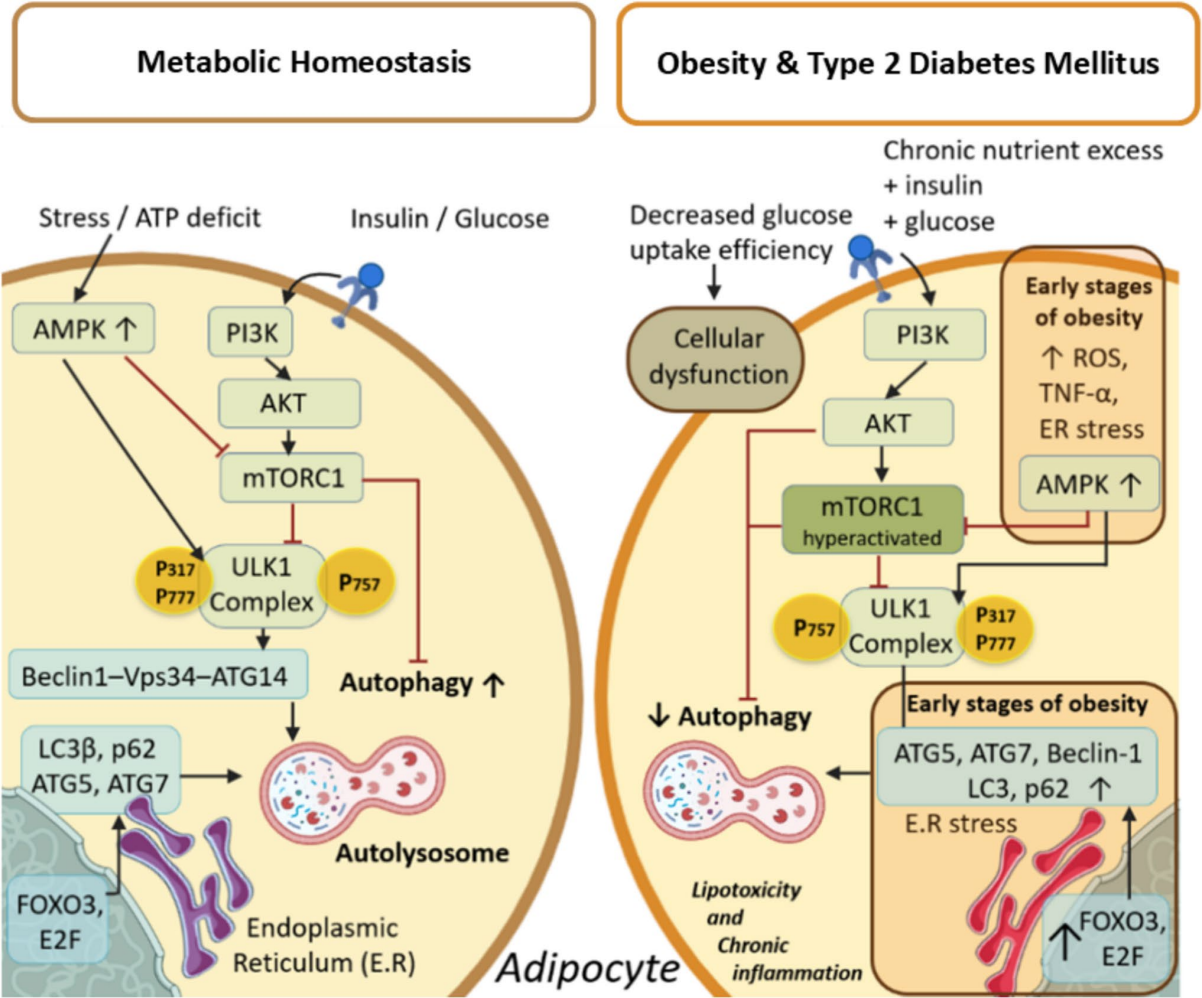
Regulation of autophagy under physiological conditions vs. obesity and T2DM. Under normal metabolic conditions, autophagy oscillates in response to nutrient availability, with transient inhibition mediated by insulin-PI3K-Akt-mTORC1 signaling and activation by AMPK during energy stress. In obesity and T2DM, chronic nutrient excess and decreased AMPK activity promote persistent mTORC1 activation and ULK1 inhibition, thereby disrupting autophagic flux. This imbalance contributes to mitochondrial dysfunction, oxidative and inflammatory stress, and impaired insulin signaling

Under physiological conditions, the mTORC1 complex pathway constitutes the central regulatory node. The insulin-PI3K-Akt-mTORC1 signaling axis transiently inhibits autophagy during feeding [8], whereas AMPK activation during energy stress promotes ULK1-dependent autophagosomes formation via the Vps34-Beclin-1-ATG14-AMBRA1 complex. This dynamic equilibrium enables coordinated changes between anabolic and catabolic states while maintaining mitochondrial integrity, redox control, and proteostasis [9, 25]. In this context, ULK1 represents a key integration point between these pathways. Therefore, when AMPK signaling predominates, ULK1 is activated and autophagosome biogenesis is initiated; and, when mTORC1 activity ptrdominates, ULK1 is inhibited, thereby reducing autophagic flux. Beyond acute kinase regulation, transcription factors of the FOXO family, particularly FOXO3, and transcription factor EB (TFEB) sustain the expression of autophagy and lysosomal-related genes, ensuring adequate degradative capacity (Fig. 1). Therefore, metabolic homeostasis depends not only on autophagosome formation but also on intact lysosomal function and effective organelle turnover [6, 21].

### Autophagy Signaling in Obesity and T2DM: Dysregulation and Uncoupling

In obesity and T2DM, chronic nutrient excess and hyperinsulinemia disrupt this regulatory network in a stage-dependent manner. In the early stages of obesity, adipose tissue expansion induces ER stress, inflammation, and hypoxia, transiently suppressing mTORC1 and activating autophagy as a compensatory response. This protective mechanism, more pronounced in visceral adipose tissue, is associated with increased expression of ATG5, ATG7, Beclin-1, LC3, and p62, regulated by transcription factors such as FOXO and E2F [25]. However, as obesity progresses and exposure to insulin and nutrients increases, mTORC1 becomes chronically overactived, thereby inhibiting autophagy and promoting lipid accumulation, lipotoxicity, and chronic inflammation in the liver and adipose tissue [6, 8].This persistent inhibition is associated with chronic sustained ULK1 supression and a reduced capacity to clear damaged organelles and maintain metabolic flexibility [21].

Furthermore, reduced AMPK activity in states of insulin resistance contributes to increased oxidative stress and inflammasome activation, leading to the release of IL-1β and TNF-α [20, 21]. In advanced stages, the PI3K-Akt axis remains chronically activated by hyperinsulinemia, further reinforcing autophagy inhibition, while impairing glucose uptake and amplifying metabolic dysfunction [19, 26].

Finally, excessive Akt signaling also induces FOXO hyperphosphorylation, promoting its nuclear exclusion and thereby limiting the expression of autophagy-related and antioxidant genes. This imbalance, characterized by mTORC1 hyperactivation and deficient AMPK-FOXO signaling, consolidates a state of chronic autophagy inhibition, that favors the accumulation of dysfunctional mitochondria, systemic inflammation, and insulin resistance [6] (Fig. 1).

Therefore, metabolic disease is characterized less by simple suppression of autophagy than by a progressive failure of coordinated organelle quality control networks, in which mTORC1-AMPK imbalance, defective mitophagy, and lysosomal dysfunction converge to sustain meta-inflammation and insulin resistance.

### Autophagy in the Central Nervous System and Metabolic Regulation

Autophagy in the central nervous system (CNS), particularly within hypothalamic nuclei, plays a critical role in the regulation of systemic energy homeostasis. Neurons in the arcuate nucleus, including pro-opiomelanocortin (POMC) and agouti-related peptide (AgRP) neurons, depend on tightly regulated autophagic activity to integrate nutrient and hormonal signals and to coordinate feeding behavior and energy expenditure {Kaushik, 2011, 21,803,288}{Kim, 2015, 26,411,686}{Bhusal, 2021, 34,910,246}.

Experimental evidence indicates that hypothalamic autophagy is dynamically regulated by nutrient availability and endocrine cues. Fasting activates neuronal autophagy through AMPK signaling, promoting intracellular lipid mobilization and supporting neuronal energy sensing, whereas nutrient excess and chronic hyperinsulinemia drive mTORC1 activation and suppress autophagic flux {Oh, 2016, 27,533,078}. In murine models, genetic disruption of autophagy in pro-opiomelanocortin (POMC) neurons leads to altered lipid sensing and impaired satiety signaling, contributing to positive energy balance and weight gain, whereas altered autophagy in AgRP neurons affects adaptive responses to fasting {Meng, 2011, 21,784,844}{Kim, 2015, 26,411,686}.

Importantly, obesity is associated with hypothalamic inflammation, endoplasmic reticulum stress, and defective autophagic flux, which together contribute to central leptin and insulin resistance {Meng, 2011, 21,784,844}{Bhusal, 2021, 34,910,246}. Conversely, impaired autophagy further disrupts neuronal responsiveness to hormonal signals, highlighting a bidirectional relationship between autophagy and neuroendocrine regulation. This impairment exacerbates systemic metabolic dysfunction by altering glucose homeostasis and energy balance. Thus, CNS autophagy represents a key integrative node linking peripheral metabolic stress with central regulatory mechanisms.

### Endocrine Regulation of Autophagy in Metabolic Homeostasis

Endocrine signals are major regulators of autophagy and critically shape its role in metabolic homeostasis. These regulatory mechanisms are frequently disrupted in metabolic disease, where endocrine imbalance contributes to context-dependent alterations in autophagic activity. Insulin is a primary inhibitor of autophagy through activation of the PI3K-Akt-mTORC1 pathway, thereby suppressing ULK1 activity and autophagosome formation {Frendo-Cumbo, 2021, 34,336,862}. In contrast, glucagon promotes autophagy, particularly in the liver, by activating AMPK and inhibiting mTORC1, thereby facilitating nutrient mobilization during fasting {Sunilkumar, 2021, 33,872,697}{Xu, 2026 #9493}. Together, these endocrine signals position autophagy as a key downstream effector of systemic metabolic regulation.

Adipokines and gut-derived hormones further modulate autophagic activity. Leptin exerts context-dependent effects and may regulate autophagy in peripheral tissues while contributing to central resistance in obesity {Hu, 2025, 40,932,169}. Adiponectin, in contrast, activates AMPK and enhances autophagic flux, thereby improving insulin sensitivity and mitochondrial function {Kubota, 2007, 17,618,856}{Ahlstrom, 2017, 28,954,814}. Additionally, incretin hormones such as GLP-1 may indirectly influence autophagy through modulation of insulin secretion and cellular stress pathways {Spezani, 2026, 41,417,476}.

In obesity and T2DM, chronic hyperinsulinemia, leptin resistance, and altered adipokine profiles contribute to sustained mTORC1 activation and impaired AMPK signaling, thereby reinforcing autophagy dysregulation across multiple tissues. These endocrine alterations underscore the importance of interpreting autophagy within a systemic hormonal context rather than as a purely cell-autonomous process.

## Evidence of Autophagy Dysregulation in Obesity and T2DM

Accumulating evidence indicates that both obesity and T2DM are associated with profound alterations in the autophagic machinery at a systemic level. Far from representing a uniform phenomenon, clinical and preclinical studies consistently support the presence of impaired autophagic flow in metabolically active organs such as adipose tissue, liver, skeletal muscle, and pancreatic β cells, with each tissue exhibiting a distinctive pattern of dysregulation [6, 19].

### Tissue Specificity and Metabolic Consequences

In adipose tissue, reduced effective autophagic flux in adipocytes and stromal cells contributes to cellular hypertrophy, ER stress, and inflammatory activation [27]. In parallel, in the liver, defects in the autophagosome-lysosome system, frequently associated with impaired TFEB signaling, compromise lipid and protein degradation, exacerbating metabolic hepatic steatosis and systemic insulin resistance [6].

Skeletal muscle and pancreatic β cells, in contrast, exhibit a critical dependence on mitochondrial quality control. In these tissues, autophagy operates within a narrow “homeostatic window”, in which both insufficient and excessive activity can compromise cellular function [21, 28]. Therefore, moderate activation of autophagy (often AMPK-dependent) preserves β-cell function, whereas chronic or uncontrolled induction may trigger apoptotic pathways [8, 21]. This duality suggests that therapeutic strategies should aim to restore context-specific balance according to the tissue and pathological stage, rather than promoting indiscriminate global activation or inhibition.

### Mitophagy and Organ Dysfunction

A distinctive feature of metabolic disease progression is the impairment of mitophagy. Alterations in the PINK1/Parkin and BNIP3/NIX signaling pathways facilitate the accumulation of dysfunctional mitochondria, increasing reactive oxygen species (ROS) production and reducing metabolic flexibility [26]. This defect is particularly evident in cardiac tissue, where it contributes to metabolic deterioration and the cardiovascular complications associated with obesity. In this context, the use of compounds such as melatonin and urolithin A have shown promising results in experimental models by restoring mitochondrial quality control mechanisms [26].

### Intersection with Immune Metabolism and Methodological Limitations

Autophagy also acts as a regulatory node in inflammatory processes. Autophagy-deficient macrophages exhibit increased activation of the NLRP3 inflammasome, resulting in increased secretion of proinflammatory cytokines such as IL-1β and IL-18. This mechanism reinforces the meta-inflammatory state observed in individuals with obesity [20].

However, interpretation of these findings in humans is limited by the predominant use of static markers (such as LC3, p62), whose levels can fluctuate according to tissue type and disease stage, and do not always reflect true degradative flux. This methodological ambiguity complicates interpretation and may account for apparently contradictory findings across tissues and patient cohorts.

Current consensus positions autophagy not as a uniformly suppressed or hyperactivated pathway, but rather as a tissue-specific maladaptive process. This dysfunction constitutes a mechanistic link between mitochondrial failure, β-cell survival, and immune homeostasis in the pathogenesis of T2DM. Despite these advances, much of the mechanistic understanding derives from experimental systems; therefore, direct evidence in chronic human metabolic diseases remains limited and represents a priority area for future research.

## Monitoring Autophagy in Metabolic Diseases: Biomarkers, Limitations and an Operational Framework

Despite growing interest in targeting autophagy as a therapeutic strategy for obesity and T2DM, significant methodological gaps continue to hinder clinical translation. Current guidelines emphasize that isolated measurement of static markers is insufficient to capture true autophagic activity, as it does not distinguish between increased autophagosome formation and impaired lysosomal degradation.

Therefore, it is recommended to evaluate autophagic flow using a combination of biomarkers and functional assays [29]. Among the most extensively studied biomarkers are LC3, p62/SQSTM1, Beclin-1, and the core autophagy genes ATG5 and ATG7, in addition to emerging candidates related to mitophagy, CMA and extracellular vesicles/exosomes.

### Basic Markers: Informative but Insufficient on their Own

The LC3 protein remains the most widely used marker for detecting autophagosomes; its conversion from the cytosolic form LC3-I to the lipidated form LC3-II reflects phagophore membrane elongation [29]. However, increased LC3-II levels do not necessarily indicate pathway activation, as they may result from impaired lysosomal fusion or defective vesicular degradation. For this reason, LC3 analysis should be complemented by assessment of p62/SQSTM, an adaptor protein degraded by autophagy, but also transcriptionally regulated by NRF2 and NF-κB pathways frequently activated during meta-inflammation [30, 31]. Therefore, isolated interpretation of LC3 or p62 is unreliable in the context of obesity and T2DM, and ex vivo functional assays using lysosomal inhibitors are required to differentiate between induction and blockage of autophagic flow [29, 32].

On the other hand, early-stage markers such as Beclin-1, ATG5, and ATG7 provide insight into pathway engagement but do not inform on final degradative capacity [9]. Altered expression of these proteins has been observed in models of insulin resistance and metabolic-associated liver disease [19]. Clinically, reduced circulating Beclin-1 levels have been associated with increased risk of atherosclerosis and poorer metabolic control in T2DM, suggesting potential utility as a complementary circulating biomarker for cardiovascular disease [33]. In this context, Grazide et al*.* demonstrated that circulating Beclin-1 and ATG5 levels are independently associated with acute myocardial infarction [34].

Moreover, circulating Beclin-1 has emerged as a potential early biomarker of kidney injury in T2DM. Decreased serum Beclin-1 levels have been linked to the progression of diabetic nephropathy, reflecting impaired autophagic activity correlated with albuminuria and the stage of kidney disease [35]. These findings suggest that Beclin-1 may reflect systemic cellular stress rather than serving as a direct measure of autophagic flux.

Thus, rather than discarding classical markers such as LC3 and p62, it may be more appropriate to consider them as anchoring biomarkers within a hierarchical framework, in which their interpretation is contingent upon complementary functional assays, lysosomal competence, and tissue context. Within this model, static markers serve as entry points for pathway interrogation, but not as standalone indicators of autophagic flux.

### Mitophagy and Organelle-specific Readings

Given the central role of mitochondrial dysfunction, lipotoxicity, and insulin resistance in metabolic diseases, mitophagy markers such as PINK1, Parkin, BNIP3, and NIX may provide more functionally relevant information than global autophagy markers. Experimental evidence indicates that modulation of these pathways improves insulin sensitivity and reduces inflammatory burden [36]. However, standardized quantification methods in human tissues remain lacking.

### Lysosomal and CMA-related Markers

Autophagic efficiency ultimately depends on lysosomal competence. It is now recognized that alterations in TFEB signaling, lysosomal enzymes activity, and LAMP2A-dependent CMA in metabolic tissues link defective degradation with lipid accumulation and reduced metabolic flexibility [37, 38]. These markers may help identify defects downstream of autophagosome formation. Nevertheless, their clinical validation in individuals with obesity or T2DM remains limited and requires well-designed translational studies.

### Extracellular Vesicles as Systemic Reporters

Biomarkers derived from circulating exosomes provide a non-invasive window into metabolic dysfunction. Several exosomal miRNAs (including miR-32-3p, miR-34a-5p, miR-122-5p, and miR-570-3p) have been associated with insulin resistance, liver injury, and β-cell stress [39, 40]. These findings suggest that exosomal miRNAs reflect systemic metabolic stress and may serve as early indicators of functional decline. However, direct mechanistic links between circulating exosomal cargo and autophagy-related pathways in humans remain limited, warranting further investigation.

Taken together, these findings underscore that no single biomarker can comprehensively capture the autophagic state. Evidence supports the integration of classic tissue markers (LC3, p62, Beclin-1, ATG5/7) with functional assessments of autophagic flux and exosomal biomarker profiling as the most robust strategy for evaluating autophagic dysfunction associated with obesity and T2DM. Implementation of such integrated panels in clinical settings may enable correlation of autophagic activity with metabolic progression and open new opportunities for personalized diagnosis and therapeutic intervention. The principal biomarkers discussed in this subsection are summarized in Table 1.

**Table 1:** Summary of autophagy biomarkers described in human obesity and T2DM

Biomarker	What it measures/Autophagy status	Strengths/Clinical utility	Limitations	References
LC3-I/LC3-II	Autophagosome formation/phagophore membrane elongation	Widely used and well characterized; allows estimation of changes in autophagic vesicles generation; applicable in adipose tissue or skeletal muscle biopsies	Elevated levels may reflect either induction or blockage of autophagy; interpretation requires flux assays or complementary markers	[29, 32]
p62/SQSTM1	Cargo recognition and delivery to autophagosomes	Serves as an indicator of autophagic clearance, accumulation correlates with metabolic dysfunction in certain human studies	Transcriptionally regulated and stress-responsive; may increase independently of impaired flux; expression varies by tissue	[19, 32, 41–44]
Beclin-1	Initiation of autophagosome assembly	Reflects early-stage autophagy activation; circulating levels correlate with cardiovascular disease (CVD) risk and kidney injury	Does not necessarily reflect downstream autophagic flux; influenced by multiple regulatory pathways	[33, 34]
ATG5, ATG7	Autophagosome elongation machinery	Indicates pathway engagement; circulating levels associated with CVD events	Tissue-specific expression; does not distinguish between flux induction and degradation blockade	[19]
PINK1/Parkin/BNIP3/NIX	Mitophagy	Reflects mitochondrial quality control; potential therapeutic targets in insulin resistance and metabolic intervention	Primarily evaluated in experimental settings; limited standardized quantification in human tissues	[36, 45]
LAMP2A/HSC70	CMA	Early indicator of metabolic dysfunction; relevant to steatosis, lipolysis, thermogenesis, and fatty acid oxidation	Limited human data; tissue-specific expression; complex flow assessment; LAMP2A quantification generally requires tissue samples (liver, adipose tissue)	[37, 38]
Exosome-derived proteins/microRNAs (e.g., miR-32-3p, miR-34a-5p)	Indirect autophagy-related signaling/systemic metabolic stress	Non-invasive; potential for longitudinal monitoring; correlates with metabolic parameters (HbA1c, insulin resistance)	Standardization of isolation and validation protocols is still required; clinical application in early stage	[39, 46]

### Operational Framework for Human Studies

Based on recent findings, we propose an operational panel integrating: (1) direct tissue assessment through biopsies; (2) functional autophagic flux assays in peripheral blood mononuclear cells (PBMCs); and (3) analysis of circulating and exosome-derived biomarkers (Table 2). This panel combines direct evaluation of metabolically active tissues such as liver, skeletal muscle, and adipose tissue, using molecular and morphological techniques (Western blotting, immunohistochemistry, electron microscopy, and RT-qPCR), with ex vivo functional assays incorporating lysosomal inhibitors to determine autophagic flux efficiency. Complementarily, peripheral blood and plasma exosome analyses provide a less invasive and potentially longitudinal strategy, employing methodologies such as ELISA, flow cytometry, immunoblotting, and quantification of Atg-related miRNAs.

**Table 2:** Panel of operational biomarkers, applicable in a translational clinical setting

Panel component	Sample/Tissue	Biomarkers	Target
Metabolic target tissue	Biopsy (Visceral/subcutaneous adipose tissue, skeletal muscle, or liver)	LC3-II/LC3-I (WB), p62 (WB/immunohistochemistry), Beclin-1, ATG5, ATG7 (mRNA and protein)	To evaluate tissue capacity to initiate autophagy, assess accumulation of stress-related markers, and confirm degradative blockade through comparison with ex vivo treatment using lysosomal inhibitors
Peripheral cells (ex vivo)	Peripheral blood mononuclear cells (PBMCs) (Peripheral blood)	Functional autophagic flux assessment with and without lysosomal inhibitors (e.g., chloroquine or bafilomycin); measurement of LC3-II, p62; evaluation of mitophagy markers (PINK1/Parkin)	Provides a minimally invasive functional approach; suitable for therapeutic monitoring and potential patient stratification
Exosome-derived proteins and circulating biomarkers	Blood (Plasma/serum)	Circulating Beclin-1 and ATG5 (ELISA); ATG-related proteins and specific miRNAs (e.g., miRs targeting ATG genes) in exosomes	Non-invasive strategy for longitudinal monitoring of metabolic dysfunction; enables correlation with HbA1c, insulin resistance and body weight
Metabolic phenotyping	Clinical and analytical data	HbA1c, HOMA-IR, lipid profile, BMI, waist circumference, inflammatory markers (TNF-α, IL-6)	To correlate molecular biomarkers of autophagy with metabolic disease severity and to validate their diagnostic and prognostic value at the patient level

**Fig. 2:**
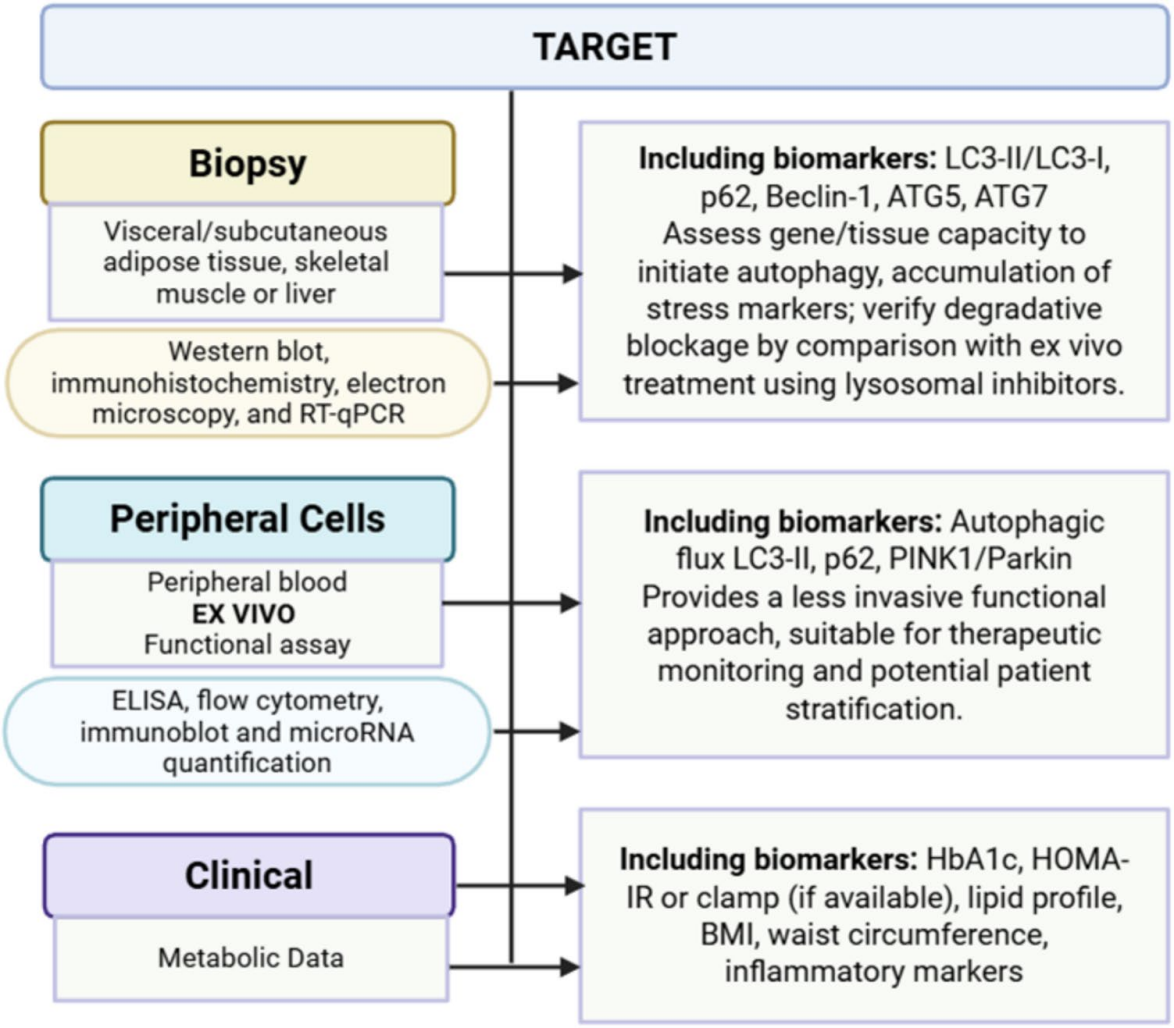
Monitoring of autophagy. Operational framework for the assessment of autophagic activity in both research and clinical results

This comprehensive approach enables correlation of cellular and molecular parameters with clinical and metabolic indicators, including HbA1c, HOMA-IR, lipid profile, and systemic inflammatory markers, thereby facilitating a more integrated assessment of the relationship between autophagic activity and the patient's metabolic status. The structure and workflow of this approach are summarized in Table 2 and visually represented in the flowchart (Fig. 2), which describes an operational circuit for monitoring autophagic activity in both research and clinical settings.

Importantly, this framework is not intended to be applied in its entirety within routine clinical practice, but rather as a modular and scalable strategy. While tissue biopsies and ex vivo assays may be primarily restricted to research settings, circulating and exosome-derived biomarkers, combined with metabolic phenotyping, represent a more immediately translatable approach for clinical implementation.

This approach resolves the apparent paradox whereby the same indicators are both limited and indispensable, such that their value lies not in their isolation, but in their integration within multilevel evaluation strategies.

### Toward a Pragmatic, Tiered Framework for Clinical Translation

While comprehensive multi-tissue and multi-omic approaches provide valuable mechanistic insight, their direct implementation in clinical settings is limited by considerations of feasibility, cost, and invasiveness. Therefore, a tiered strategy may offer a more realistic path toward clinical translation.

At a first level, within routine clinical practice, non-invasive biomarkers such as circulating Beclin-1, ATG5, and exosome-derived miRNAs, combined with standard metabolic parameters (HbA1c, HOMA-IR, lipid profile), may provide an initial assessment of autophagy-related alterations.

At a second level, functional studies, such as ex vivo assays in PBMCs using lysosomal inhibitors, can provide a minimally invasive estimation of autophagic flux, suitable for longitudinal monitoring and therapeutic evaluation.

At a third level, in-depth phenotypic characterization, including tissue biopsies and advanced techniques such as electron microscopy or flux assays in primary cells, should be reserved for mechanistic studies or selected clinical cohorts.

This stratified approach balances biological resolution with clinical feasibility and may facilitate the progressive integration of autophagy biomarkers into metabolic disease management.

## Emerging Therapeutic Strategies: Autophagy Modulators, Natural Compounds, and Drugs in Development

The therapeutic modulation of autophagy remains under critical evaluation. Current strategies range from lifestyle interventions [47, 48], to widely used pharmacological agents such as metformin [49] and thiazolidinediones (TZDs) [50], as well as natural or nutraceutical compounds including resveratrol [51]. In addition, several next-generation molecules are currently under experimental investigation [1].

**Fig. 3:**
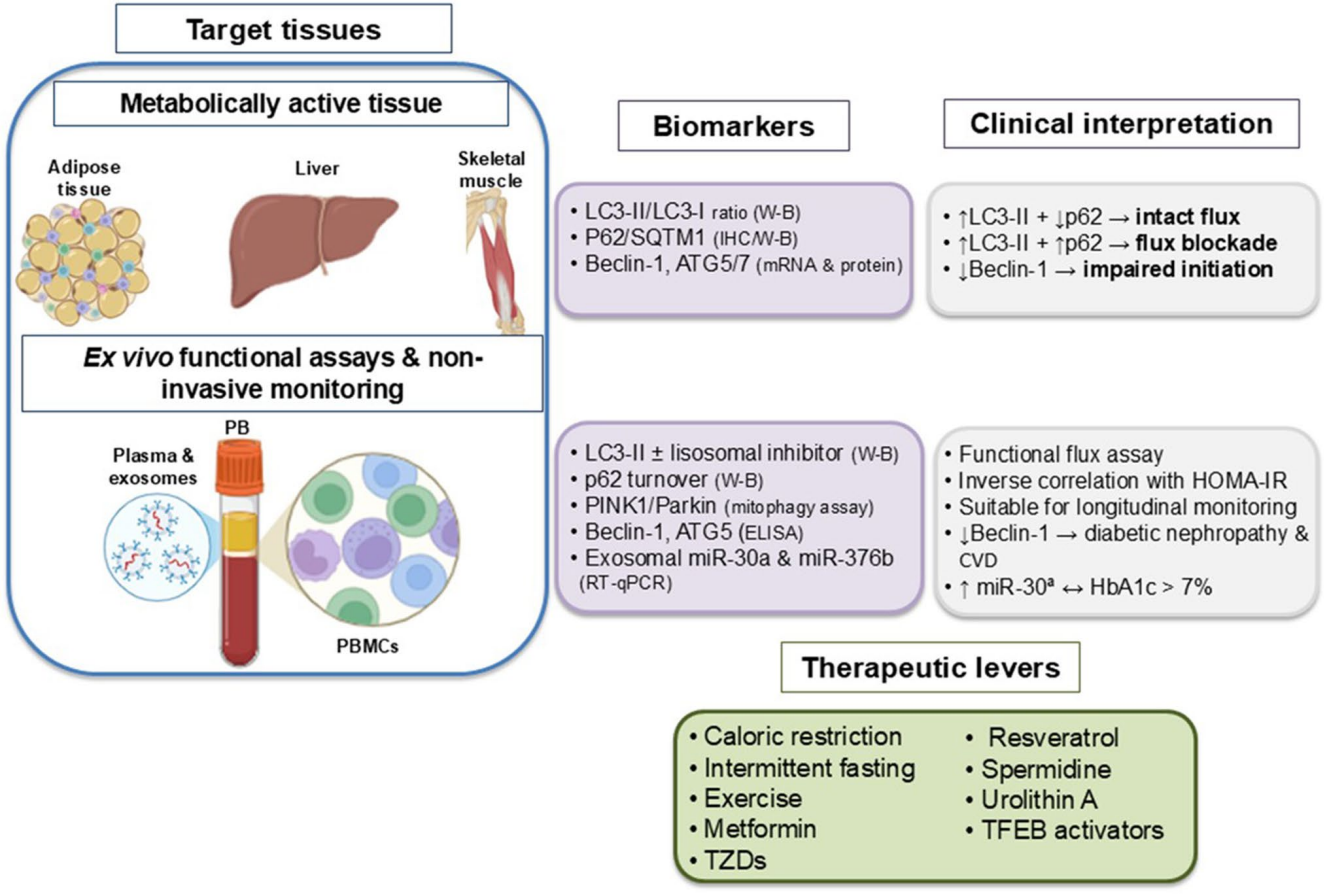
Proposed translational panel for the assessment of autophagy in patients with obesity and/or T2DM. Evaluation is stratified across three levels: (1) metabolically active tissue (adipose tissue, skeletal muscle, or liver) to quantify markers of autophagic initiation and flux; (2) peripheral blood mononuclear cells (PBMCs) for ex vivo functional assays; and (3) plasma and circulating exosomes for noninvasive monitoring. Interpretation is integrated with clinical parameters (HbA1c, HOMA-IR) to support risk stratification and evaluation therapeutic response. CVD, cardiovascular disease; IHC, immunohistochemistry; WB, western blot

Strategies acting directly or indirectly on key regulatory nodes (mTORC1, AMPK, ULK1, mitophagy) are being investigated for their ability to influence lipid handling, insulin sensitivity, adiposity, and inflammatory pathways, primarily in preclinical models. Figure 3 provides a schematic overview of these therapeutic strategies, their molecular targets, and the corresponding biomarkers used to monitor therapeutic response. The following sections critically examine their mechanisms of action and the available clinical evidence.

### Lifestyle Interventions: Physiological Activators

Caloric restriction and intermittent fasting are established as effective non-pharmacological strategies for inducing physiological autophagy, primarily through AMPK activation and mTORC1 inhibition/These interventions are associated with reductions in adiposity and improvements in glucose metabolism in humans [52].

Similarly, physical exercise enhances autophagy, especially in skeletal muscle and adipose tissue, thereby reducing ectopic lipid accumulation [53]. Although considered first-line metabolic interventions, current literature emphasizes that both the magnitude of the stimulus and tissue-specific responses are heterogeneous. A standardized "optimal protocol" for clinical implementations in T2DM or obesity has yet to be defined [19].

### Current Pharmacotherapy: Indirect Effects on Autophagy

Metformin, the established first-line therapy for T2DM, exerts metabolic effects extending beyond suppression of hepatic gluconeogenesis. It functions as an energy modulator and regulator of autophagy [54]. By inhibiting mitochondrial complex I, metformin increases the intracellular AMP/ATP ratio, thereby activating the AMPK-ULK1 pathway and inhibiting mTORC1; which collectively enhances autophagic flux [54].

Furthermore, AMPK activation mediated by metformin represses the expression of gluconeogenic genes such as PEPCK and G6Pase, reducing hepatic glucose production. In adipose and skeletal muscle tissue, it promotes fatty acid oxidation and GLUT4 translocation, thereby optimizing insulin sensitivity [55]. Notably, the net autophagic effects of metformin are tissue-specific and depend on the signaling context of individual organs and tissues [49].

Evidence from animal models further support this mechanism. Metformin has been shown to activate autophagy not only via AMPK/mTOR modulation but also through inhibition TGF-β1/Smad3 signaling, promoting weight reduction and improved lipid metabolism in diet-induced obesity models [56]. Likewise, in diabetic rats, its ability to modulate autophagy through downregulation of hypoxia-inducible factor (HIF)−1α underscores its therapeutic relevance in T2DM [57].

TZDs, including pioglitazone and rosiglitazone, are widely prescribed in the treatment of T2DM because of their ability to improve insulin sensitivity mediated by activation of peroxisome proliferator-activated receptor-gamma (PPAR-γ) [58]. These agents increase mitochondrial mass and the expression of genes involved in mitochondrial dynamics and fatty acid oxidation [50]. They also influence adipocyte biology by modulating autophagy during browning and adipogenesis processes, suggesting pleiotropic and context-dependent metabolic effects [8].

### mTOR Inhibitors: from the Laboratory to Clinical Caution

Rapamycin represents the prototype mTOR inhibitor. Its mechanism of action is based on binding to the FKBP12-mTORC1 complex, inhibiting its kinase activity and releasing ULK1 suppression, thereby inducing robust activation of autophagic flux. In cellular models, rapamycin has been observed to reduce ROS production and mitigate the inflammatory response to hyperglycemia conditions in human glomerular endothelial cells [59].

However, translation into metabolic clinical practice is complex due to tissue-specific effects. Although rapamycin increases autophagic activity in both human and murine pancreatic islets, it also reduces insulin secretion, suggesting that excessive autophagy induction may have deleterious effects on β-cell function depending on dose and duration of exposure [60]. On the other hand, chronic administration in humans is associated with adverse metabolic and immunosuppressive effects, limiting its systemic application [8]. Consequently, current research is exploring intermittent rapamycin regimens in murine models, which appear to preserve glucose homeostasis and may offer a safer alternative to continuous dosing [61].

To optimized pharmacokinetic profiles, rapamycin analogues (raparalogs) such as everolimus and temsirolimus have been developed. Although they share rapamycin’s mechanism of action, their metabolic effects in obesity and T2DM models are heterogeneous; in this sense, protocols based on low/intermittent doses, or combinations atrategies, have demonstrated metabolic benefits, whereas continuous or high-dose administration may induce glucose intolerance, hyperlipidemia, or impaired Akt signaling [62, 63].

Second-generation mTOR inhibitors, such as Torin-1, PP242 (Torkinib), and AZD8055 (Vistusertib), act by dually blocking mTORC1/mTORC2 through competitive inhibition of the ATP-binding site. Evidence from tumor cell lines indicates that these compounds induce a more profound activation of autophagy than rapamycin, as demonstrated by the conversion of LC3-I to LC3-II and the downregulation of p62/SQSTM1, consistent with enhanced autophagic flux. However, such marked activation may result in dysfunctional autophagy and promote cell survival (as observed with AZD8055) or culminate in excessive mitophagy leading to apoptotic cell death (as with PP242) [64, 65].

Finally, dual PI3K/mTOR inhibitors such as BEZ235 (Dactolisib) and GDC-0980 (Apitolisib) have shown potential in models of insulin; however their clinical application is severely limited by adverse metabolic effect, including hyperglycemia and insulin feedback activation, which restrict their therapeutic use [66].

It is important to emphasize that most of these inhibitors were originally developed for oncological indications; therefore, evidence regarding their safety and metabolic efficacy in humans remains limited and fragmented. The well-documented association between systemic mTOR inhibition and the development of dyslipidemia and impaired insulin signaling currently precludes the consideration of these compounds as chronic therapies for obesity or T2DM.

### Natural Compounds with Evidence in Clinical Trials

Certain naturally occurring compounds with pro-autophagic activity have gained attention based on robust preclinical data and emerging early-phase clinical trials. Among these, spermidine, a dietary polyamine, stimulates autophagy through epigenetic mechanisms and has been associated with improvements in metabolic and mitochondrial biomarkers in experimental systems and as well as in early-phase human studies [67].

Similarly, urolithin A, a microbiota-derived metabolite generated from dietary ellagitannins, activates mitophagy and enhances mitochondrial function. Early clinical studies have demonstrated its safety and its capacity to improve biomarkers of mitochondrial function in humans, positioning it as a promising translational candidate for metabolic diseases [26, 68].

In the same context, resveratrol has been reported to promote autophagy through activation of SIRT1, inhibition of mTOR, and subsequent improvement of lipid metabolism and adiposity [69]. In animal models, resveratrol has been shown to induce autophagy while reducing apoptosis in diabetic mice and podocytes through suppression of microRNA-383-5p [70].

### The Future: Precision and New Targets

Research is now turning towards new small molecules such as MSL-7, which enhance autophagy by activating TFEB without directly inhibiting mTOR, thereby potentially avoiding the metabolic adverse effects of the latter. In preclinical models, MSL-7 has been reported to exert its effects by inducing calcineurin-mediated TFEB dephosphorylation and promoting inflammasome activation in obese and diet-induced obese mice [71].

Looking forward, several emerging strategies may further refine autophagy-based therapeutic approaches. Gene-editing technologies, such as CRISPR/Cas9, offer the possibility of directly targeting key genes related to autophagy, thereby opening new therapeutic avenues, particularly for disorders with genetic alterations affecting autophagy regulation [72, 73].

In parallel, advances in nanotechnology and targeted delivery systems have catalyzed the development of platforms capable of delivering autophagy modulators with greater precision and selectivity, thereby optimizing bioavailability and minimizing systemic adverse effects [74]. Nanomedicine-based systems have demonstrated the ability to modulate autophagy, either by inducing or inhibiting it, in several experimental models, including cancer, and represent a potential frontier for future clinical translation [75]. Moreover, autophagy-modulating nanoparticles may serve a dual function: first, to enhance therapeutic efficacy through targeted action, and second, to function as early biomarkers of toxicity or cellular response, thereby supporting the development of more personalized and safer diagnostic and therapeutic strategies [76]. Nevertheless, these approaches remain largely experimental and face significant challenges, including tissue-specific delivery, off-target effects, long-term safety concerns, and regulatory challenges.

Although autophagy is considered an emerging therapeutic target in metabolic diseases, no currently approved treatment for obesity or T2DM exerts its primary therapeutic effect through direct modulation of autophagy. However, several widely used agents, including metformin, thiazolidinediones, and resveratrol, indirectly influence autophagic pathways. Larger clinical trials are anticipated in the coming years to evaluate selective modulation of autophagy as a potential metabolic intervention. Current advances in autophagy modulation as a therapeutic target are summarized in Table 3.

**Table 3:** Advances in the modulation of autophagy as a therapeutic target in obesity and T2DM

Category/Treatment	Main mechanism of autophagy	Metabolic or therapeutic effects	References
Caloric restriction	↑AMPK, ↓mTORC1 → physiological activation of autophagy	↑Glucose metabolism, ↓adiposity	[47, 52]
Intermittent Fasting	↑AMPK, regulation of cellular energy	↓Body fat, ↑insulin sensitivity	[47, 48]
Physical exercise	↑Autophagy in muscle and adipose tissue	↑Fat oxidation, ↑systemic metabolism	[47, 48]
Metformin	Complex I inhibition → ↑AMP/ATP → ↑AMPK → ↓mTORC1 → ↑ULK1	↑Autophagy, ↑insulin sensitivity, ↓hepatic gluconeogenesis	[49, 54–56]
Rapamycin	↓mTORC1 through inhibition of FKBP12 → activation of autophagy	↓ROS and inflammation; risk of adverse metabolic effects with chronic use	[8, 59–61]
Everolimus/Temsirolimus	mTORC1 inhibitors (rapamycin analogues)	↑Metabolic at low/intermittent doses; risk of glucose intolerance with chronic treatment	[62, 63]
Torin-1, PP242 (Torkinib), AZD8055 (Vistusertib)	Dual mTORC1/mTORC2 inhibitors (ATP-competitive)	↑Better autophagy than rapamycin; apoptosis or dysfunctional autophagy	[64, 65]
BEZ235 (Dactolisib), GDC-0980 (Apitolisib)	PI3K/mTOR inhibitor	Potential for insulin resistance; limited by hyperglycemic effects	[66]
TZD (pioglitazone, rosiglitazone)	Activation of PPAR-γ → ↑mitochondrial autophagy and lipid dynamics	↑Insulin sensitivity, modulates adipogenesis and “browning”	[8, 50, 58]
Spermidine	↑ Proautophagic epigenetic genes	↑ Mitophagy and metabolic health	[67]
Urolithin A	↑ mitophagy via mitochondrial signaling	↑ Mitochondrial and metabolic function; safe in clinical trials	[26, 68]
Resveratrol	↑SIRT1 → ↓mTOR → ↑autophagy	↑ Lipid metabolism, reduces apoptosis in T2DM	[1, 51, 69, 70]
MSL-7	↑TFEB via calcineurin-mediated dephosphorylation (without mTOR inhibition)	↑ Metabolic profile and ↑ autophagy in diet-induced obesity	[71]
Gene editing (CRISPR/Cas9)	Targeted modification of autophagy regulatory genes (ATG, Beclin-1, ULK1) → restoration of autophagy flux	Potential to correct genetic defects that compromise autophagy; opens new avenues for precision therapy	[72, 73]
Nanomedicine/Autophagy-Modulating Nanoparticles	Targeted release of autophagy modulators to specific tissues; nanomaterial-organelle interaction to ↑ or ↓ autophagy	↑ Bioavailability and therapeutic specificity; potential use as biomarkers of toxicity or cellular response	[75, 76]

## Conclusions and Future Directions

Current evidence confirms that autophagy represents a central node in metabolic homeostasis, integrating the energetic, inflammatory, and oxidative disturbances that characterize obesity and T2DM. However, this review emphasizes that autophagic dysfunction should not be interpreted as a simple, uniform suppression; rather, it reflects a tissue-specific maladaptation that depends on the stage and context of the disease.

This distinction is crucial for clinical practice: excessive or inappropriate activation may compromise β-cell function, promote skeletal muscle wasting, or trigger maladaptive responses in other metabolically active tissues. Accordingly, the therapeutic objective should not be indiscriminate stimulation, but the restoration of an autophagic flux that is appropriate to the specific biological and clinical context.

Advancement toward precision medicine in this field requires moving beyond the use of isolated static markers, which have yielded heterogeneous and sometimes contradictory results in the literature. We propose that effective clinical translation requires a comprehensive operational framework that combines:

1. Assessment of core biomarkers (LC3, p62, Beclin-1) in combination with functional autophagic flux assays.

2. Incorporation of mitophagy markers and exosomal cargo indicators to enable non-invasive systemic monitoring.

3. Close correlation of these molecular parameters with the patient's metabolic phenotyping (HbA1c, HOMA-IR and meta-inflammation indicators).

From a therapeutic standpoint, although preclinical findings are robust, clinical evidence remains limited and heterogeneous. While lifestyle interventions and agents such as metformin exert indirect effects on autophagic pathway, the future likely lies in the development of more selective modulators. Emerging strategies such as nanomedicine-based targeted delivery systems and gene editing technologies (CRISPR/Cas9) offer the potential to correct specific defects within target tissues, thereby minimizing systemic toxicity and optimizing bioavailability.

Ultimately, for autophagy to be consolidated as a viable therapeutic target, successful clinical translation will depend fundamentally on our ability to integrate mechanistic findings with precision diagnostic strategies. To achieve this goal, it is imperative to address critical challenges, including the standardization of protocols for the functional assessment of autophagic flux in humans and the implementation of controlled clinical trials to validate the safety and efficacy of novel modulators capable of restoring cellular homeostasis without disrupting systemic metabolic balance.

Future clinical trials should aim to validate this tiered biomarker framework through longitudinal study designs that integrate functional PBMC-based assays, exosomal miRNA profiling, and metabolic phenotyping, with the goal of establishing autophagy-centered precision medicine strategies for obesity and T2DM.

## Key References

- Cheong LYT, Saipuljumri EN, Loi GWZ, Zeng J, Lo CH. Autolysosomal Dysfunction in Obesity-induced Metabolic Inflammation and Related Disorders. *Curr Obes Rep. 2025;14(1):43*. 10.1007/s13679-025–00638-8.
  - *Of outstanding importance*
    This recent review highlights lysosomal dysfunction as a central bottleneck in autophagic flux during obesity-associated metabolic inflammation, emphasizing degradative impairment rather than defective initiation as a key pathogenic mechanism.
- Kim S, Choi C, Son Y, Lee J, Joo S, Lee YH. BNIP3-mediated mitophagy in macrophages regulates obesity-induced adipose tissue metaflammation. Autophagy. 2025;21(9):2009–27. 10.1080/15548627.2025.2487035.
  - *Of outstanding importance*
    This mechanistic study establishes a direct link between macrophage mitophagy and adipose tissue inflammation in obesity, supporting organelle-specific quality control as a determinant of immunometabolic dysfunction.
- Tang S, Hao D, Ma W, Liu L, Gao J, Yao P, et al. Dysfunctional Mitochondria Clearance in Situ: Mitophagy in Obesity and Diabetes-Associated Cardiometabolic Diseases. *Diabetes & metabolism journal. 2024;48(4):503–17* 10.4093/dmj.2023.0213.
  - *Of outstanding importance*
    This review synthesizes current evidence on mitophagy in cardiometabolic disease, underscoring mitochondrial quality control as a translational target in obesity and T2DM.
- Munera-Rodriguez AM, Leiva-Castro C, Reina-Perez I, Benitez-Marquez JM, Palomares F, Lopez-Enriquez S. The Role of Autophagy in Inflammatory Diseases: Challenges and Therapeutic Potential. *Inflamm Bowel Dis. 2026 Mar 1;32(3):562–571.* 10.1093/ibd/izaf279.
  - *Of importance*
    This review discusses context-dependent autophagy regulation in chronic inflammatory diseases and provides conceptual insight into the immunometabolic crosstalk relevant to obesity and T2DM.
- Xing L, Mondesir R, Glasstetter LM, Zhu XY, Lu B, Al Saeedi M, et al. The Impact of Obesity on Autophagy in Human Adipose-Derived Mesenchymal Stromal Cells. *Cell Transplant*. 2025 Jan-Dec:34:9,636,897,251,323,339. 10.1177/09636897251323339.
  - *Of importance*
    This human-based study provides translational evidence of obesity-associated alterations in autophagic pathways within adipose-derived stromal cells.
- Choi YJ, Yun SH, Yu J, Mun Y, Lee W, Park CJ, et al. Chaperone-mediated autophagy dysregulation during aging impairs hepatic fatty acid oxidation via accumulation of NCoR1. *Mol Metab. 2023;76:101,784.* 10.1016/j.molmet.2023.101784.
  - *Of importance*
    This study demonstrates the metabolic relevance of chaperone-mediated autophagy in hepatic lipid handling, extending the autophagy framework beyond macroautophagy.

## Data Availability

Not applicable.
